# Life-threatening hypersplenism due to idiopathic portal hypertension in early childhood: case report and review of the literature

**DOI:** 10.1186/1471-230X-10-122

**Published:** 2010-10-20

**Authors:** Jan Däbritz, Jennifer Worch, Ulrike Materna, Bernward Koch, Gabriele Koehler, Christina Duck, Michael C Frühwald, Dirk Foell

**Affiliations:** 1 Department of General Pediatrics, University Children's Hospital Muenster, Münster, Germany; 2 Department of Pediatric Hematology and Oncology, University Children's Hospital Muenster, Münster, Germany; 3 Department of Radiology, University Hospital Muenster, Münster, Germany; 4 Department of Pediatric Surgery, University Hospital Muenster, Münster, Germany; 5 Department of Pathology, University Hospital Muenster, Münster, Germany

## Abstract

**Background:**

Idiopathic portal hypertension (IPH) is a disorder of unknown etiology and is characterized clinically by portal hypertension, splenomegaly, and hypersplenism accompanied by pancytopenia. This study evaluates the pathogenic concept of the disease by a systematic review of the literature and illustrates novel pathologic and laboratory findings.

**Case Presentation:**

We report the first case of uncontrolled splenic hyperperfusion and enlargement with subsequent hypersplenism leading to life-threatening complications of IPH in infancy and emergent splenectomy.

**Conclusions:**

Our results suggest that splenic NO and VCAM-1, rather than ET-1, have a significant impact on the development of IPH, even at a very early stage of disease. The success of surgical interventions targeting the splenic hyperperfusion suggests that the primary defect in the regulation of splenic blood flow seems to be crucial for the development of IPH. Thus, beside other treatment options splenectomy needs to be considered as a prime therapeutic option for IPH.

## Background

Non-cirrhotic portal hypertension (NCPH) comprises a group of diseases with increased portal pressure in the absence of cirrhosis, most of which have portal hypertension as a late manifestation of the disease. Common causes of NCPH include extrahepatic portal venous obstruction, non-cirrhotic portal fibrosis or idiopathic portal hypertension (IPH) [1]. IPH is a rare disorder, characterized clinically by portal hypertension, splenomegaly, and hypersplenism accompanied by pancytopenia. Intrahepatic or prehepatic lesions are generally vascular, either in the portal vein, its branches or in the perisinusoidal area ('obliterative portovenopathy') and cause an increase in portal pressure.

The underlying etiology and pathogenesis are poorly understood. It has been proposed that infectious, toxic (exposure to trace metals or chemicals), immunological and immunogenetic factors may play a role [1]. As hepatosplenomegaly is the leading symptom, other causes of organomegaly such as infections, malignancy, malformations and metabolic disorders, must be ruled out. In particular, childhood onset points to inherited conditions involved in early hepatic fibrosis, with special emphasis on storage disorders. On the other hand, IPH is not associated with hepatic cirrhosis but portal vein thrombosis, non-thrombotic causes, and parenchymal atrophy of the liver secondary to portal malperfusion [1,2]. Although the etiology remains obscure, either presinusoidal or extrahepatic vasculopathy lead to elevated intrahepatic portal resistance [3-5]. The overall prognosis is generally benign, although catastrophic cases related to bleeding from esophageal varices may occur [4,6,7].

Till this time, no cases have been described in children under the age of 10 years. Here we report of a boy who developed mild hepatomegaly and severe splenomegaly at 8 months of age. Following emergency splenectomy the child improved rapidly, and all symptoms of the previous illness disappeared including signs of portal hypertension. This is the first case of catastrophic IPH in early childhood, and to our knowledge the first report of disease remission in childhood by surgical means. This points to the spleen as the origin for disease progression in IPH patients. We present a detailed diagnostic work-up for this unusual case and provide a comprehensive review of the literature.

## Case Presentation

### Clinical Presentation

A progressive splenomegaly was observed in an otherwise healthy 8 months-old Turkish infant during a pediatric routine examination. The boy's parents are non-consanguineous and healthy, as is his 6-year old brother. The patient's perinatal and neonatal history was unremarkable. An acute infection was initially suspected due to elevated EBV and CMV antibody titers, but a worsening clinical condition with progressive pancytopenia and fever prompted re-evaluation at the age of 13 months. An open liver biopsy, standard bone marrow smear and trephine biopsy demonstrated no features of a metabolic disorder, hematological or immunological disease, infection, hepatic disorder (except for a mild secondary hepatitis) or malignancy.

**Figure 1:**
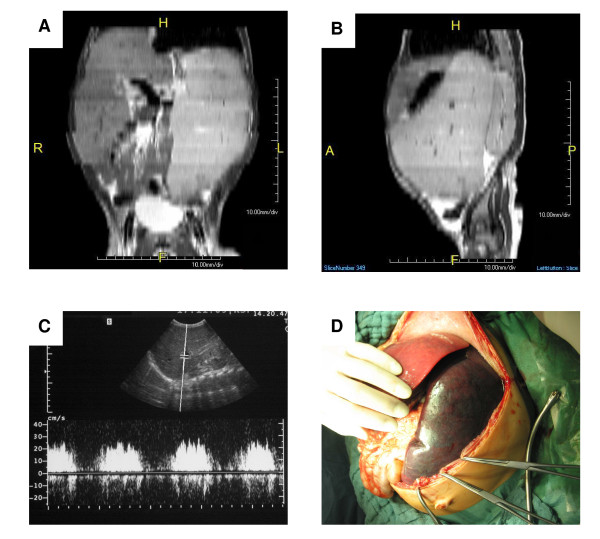
**Massive hepatosplenomegaly before splenectomy**. A) Magnetic resonance imaging (MRI) revealed enlargement of especially the spleen on axial projection. The splenic enlargement resulted in a compression of the bladder (H = Head, F = Foot, R = Right, L = Left). B) On sagittal MRI projection, compression of the left kidney was seen. The compression of other intraabdominal organs impeded alimentation (H = Head, F = Foot, A = Anterior, P = Posterior). C) On Doppler sonography, the portal vein was relatively prominent with a width of 1 cm. Portal vein blood flow was markedly increased. D) During surgery, the extent of splenomegaly was documented. The organ had a weight of 1.2 kg.

Over the following 5 months, the boy was not thriving and progression of splenomegaly was obvious by palpation and increasing abdominal girth. Abdominal ultrasound confirmed these findings: the liver size was 13.7 cm and the spleen measured 19.1 cm without any findings of abnormal vessel variations and patent hepatic veins. The portal vein was relatively prominent with a width of 1 cm, which in combination with markedly increased blood flow indicates a hyperdynamic situation (Figure 1C). We concluded that these signs were consistent with increased portal pressure. Gastroscopy confirmed congestive gastropathy grade I. The combination of findings led to the diagnosis of idiopathic portal hypertension. We decided against measuring of portal wedge pressure or partial splenic embolization due to the significant procedure associated risks in this critically ill child. Magnetic resonance imaging of the abdomen showed a dramatic enlargement of the spleen with compression of the neighboring organs (Figure 1A, B). On scintigraphic imaging with In-111-oxinate tagged platelets, liver and spleen exhibited significant pooling indicating severe sequestration in these organs. The boy received prophylactic immunization for suspected splenic dysfunction.

During the next week the child's clinical condition deteriorated further. He developed fever (despite antibiotic therapy) with no signs for inflammation, severe pancytopenia without adequate response to periodic transfusions (platelets, erythrocytes), hemorrhagic diathesis (prolonged bleeding time) and symptoms due to compression of the bordering organs (dyspnea, refusal to take food, and urinary retention). Due to his life-threatening condition splenectomy was performed, which passed without any complications. During the surgical procedure a second liver biopsy was taken, the spleen was explanted, and small accessory spleen was left *in situ *(Figure 1D). The postoperative course was uneventful and the boy's condition improved rapidly. He was discharged with an antibiotic prophylaxis and beta-blocker medication to decrease portal pressure. When last seen one and a half years after surgery, he was healthy and thriving and on abdominal ultrasound his liver size had significantly reduced in size. Furthermore, on repeated upper endoscopy the signs of hypertensive gastropathy disappeared. Beta-blocker medication was discontinued without complication.

**Figure 2:**
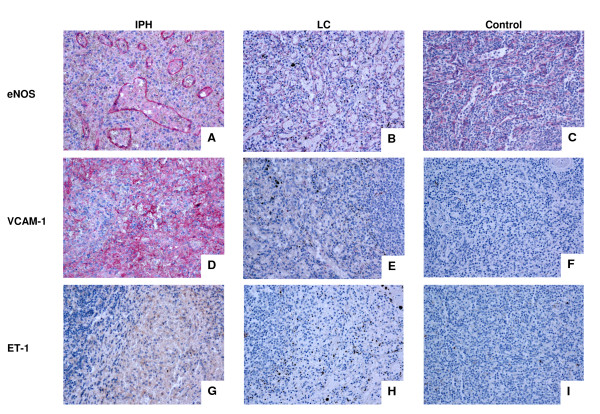
**Immunohistochemical analysis of liver and spleen at splenectomy**. There is strong staining for eNOS especially in the endothelium of the spleen of the IPH patient (A). In contrast, there is no detectable eNOS expression in the liver of the patient (not shown), in the spleen of a patient with liver cirrhosis (B) and in a healthy control (C). There is a strong expression of VCAM-1 in the spleen of our patient (D), whereas the expression is not restricted to endothelial cells but also found on stromal cells of myeloid origin. VCAM-1 staining is negative in hepatic tissue of our patient (not shown) and there was also no staining in splenic tissue of a patient with liver cirrhosis (E) and in healthy spleens obtained after trauma surgery (F). ET-1 is negative both in the spleen (G) and in the liver (not shown) of our patient, as well as in the splenic tissue of a patient with liver cirrhosis (H) and a healthy control (I). IPH, idiopathic portal hypertension; LC, liver cirrhosis, eNOS, endothelial nitric oxide synthase; VCAM-1, vascular endothelial adhesion molecule-1; ET-1, endothelin-1; Original magnifications ×200.

Histopathologic examination of the spleen and liver revealed extreme hyperplasia of the lymphatic tissue within the white pulp of the spleen (splenadenoma), sinusoid ectasia especially in the spleen and expansion of the portal tracts without further hepatic pathology. For immunohistochemistry tissue samples were frozen in liquid nitrogen and stored at -80°C until use or fixed in buffered 4% formaldehyde and routinely embedded in paraffin. Immunohistochemical studies were performed with the previously described primary antibodies recognizing ET-1 (monoclonal, mouse, alpha-Diagnostics, Germany), eNOS (polyclonal, rabbit, alpha-Diagnostics, Germany), and anti-VCAM-1 (monoclonal, mouse, BD PharMingen, Germany). In brief, after de-paraffinization, endogenous peroxidase activity was blocked with 3% hydrogen peroxide in absolute methanol for 10 min at room temperature. After incubation with the primary antibody for 12 hours at room temperature, sections were exposed to the secondary biotin-conjugated goat anti-rabbit or goat anti-mouse IgG. This was followed by amplification using system SAB (LSAB kits, Dako, Glostrup, Denmark) with 3,3'-diaminobenzidine hydrochloride. Sections were counterstained with Ehrlich hematoxylin. Immunohistochemical staining for endothelial nitric oxide synthetase (eNOS) and vascular endothelial adhesion molecule-1 (VCAM-1) showed extensive expression in the spleen but not the liver, while endothelin-1 (ET-1) staining was weak (Figure 2). In contrast, there was no detectable eNOS, VCAM-1, and ET-1 expression in the spleen of a patient with liver cirrhosis (chronic infection with hepatitis C virus) and in a healthy control (traumatic splenic rupture). Age, gender, splenic weight and size are summarized in Table 1.

**Table 1:** Main Features of the Cases Studied.

	n	Sex	Age	Splenic weight	Splenic size
IPH	1	M	20 mo	1,160 g	24.0 × 12.0 × 8.0 cm
LC	1	M	51 yr	750 g	17.0 × 15.0 × 7.0 cm
Control	1	F	46yr	94 g	9.3 × 6.8 × 4.0 cm

M, male; F, female; IPH, idiopathic portal hypertension; LC, liver cirrhosis

Serum levels of VCAM-1 and ET-1 were measured using ELISA kits according to the manufacturer's instructions (VCAM-1, Human VCAM-1 ELISA KIT, Chemicon International, Germany; ET-1, QuantiGlo, Human Endothelin-1 Immunoassay, R&D Systems, Minneapolis, MN, USA). VCAM-1 concentration was initially markedly elevated in serum (11,300 ng/ml, normal range 675 - 1,690 ng/ml), while ET-1 was normal. One year after surgery, VCAM-1 levels in serum decreased to near normal. Due to the excellent clinical course of the disease, no further measurements were performed.

### Splenectomy in IPH - Review of the Literature

While some reports of IPH in childhood exist, those published so far were of patients with a much milder disease course [6]. In our review of the literature (Table 2), a total number of 14 patients could be identified in whom IPH was diagnosed and splenectomy was performed [7-19]. None of these patients was younger than 20 years (median age 30 years, range 20-65) when presenting with symptoms. There is a male predominance with only one female patient out of 14. Our patient (age at presentation 8 months) was also male. All patients were found to have splenomegaly and pancytopenia. Gastric or esophageal varices were present in nine patients. One patient had recurrent hepatic encephalopathy for one year. Abnormal liver function tests were reported in four patients. Liver transplantation was performed in one patient at the same time as splenectomy. Histopathological features of IPH were seen in all patients, whereas portal fibrosis was explicitly reported in nine patients, vascular changes in four patients and signs of (reactive) hepatitis with inflammatory cell infiltration in five patients.

**Table 2:** Case Reports of Splenectomy In Idiopathic Portal Hypertension.

Report	Age (yrs), Sex	Dx	Splenomegaly	Thrombocytopenia	Leucopenia	Gastric varices	Esophageal varices	Encephalopathy	Ab-normal Liver function tests	Liver transplantation	Portal fibrosis	Vascular changes	Inflammation/heaptitis	Follow-up	Recovery
Upshaw (1979) [19]	46, M	AI	yes	yes	yes	no	yes	no	no	no	yes	no	no	12 yr	yes
Lindor (1987) [18]	65, M	Ly	yes	yes	yes	no	yes	no	(yes)	no	no	no	no	14 mo	yes
Petrowsky (1997) [8]	30, M	None	yes	yes	yes	no	yes	no	yes	yes	yes	yes	no	26 mo	yes
Sasajima (2006) [10]	53, F	RA	yes	yes	no	yes	no	no	yes	no	no	yes	yes	12 mo	yes
Ohta (2001) [11]	59, M	None	NA	NA	NA	NA	NA	yes	NA	no	yes	no	no	NA	yes
Inagaki (2000) [12]	38, M	SLE	yes	yes	yes	no	yes	no	yes	no	yes	no	yes	24 mo	yes
Ziarkiewicz (2004) [9]	21, M	None	yes	yes	yes	no	no	no	NA	no	yes	yes	no	6 mo	yes
Bernard (1995) [14]	20, M	None	yes	yes	yes	no	no	no	NA	yes	yes	no	no	14 yr	yes
Yoshimura (1994) [15]	30, M	RTx	yes	yes	yes	no	yes	no	no	no	yes	no	yes	6 yr	yes
Yoshimura (1994) [15]	22, M	RTx	yes	yes	yes	no	yes	no	no	no	yes	no	no	26 mo	yes
Tsujimura (1987) [16]	NA	PHI	NA	NA	NA	NA	NA	no	no	no	no	yes	yes	NA	no
Babbs (1991) [17]	30, M	IgAN	yes	no	no	no	yes	no	no	no	no	no	no	30 mo	yes
Noël (1995) [13]	36, M	RTx	yes	yes	yes	no	yes	no	no	no	yes	no	no	12 mo	yes
Treška (2005) [7]	20, M	None	yes	yes	yes	no	no	no	no	no	no	no	yes	10 mo	yes
Our Case	0.7, M	None	yes	yes	yes	no	no	no	no	no	no	no	no	18 mo	yes

M, Male; F, Female; Dx, Accompanying Disease; AI, Arsenic Ingestion; Ly, Lymphoma; RA, Rheumatoid Arthritis; SLE, Systemic Lupus Erythematosus; RTx, Renal Transplantation; PHI, Polyclonal Hyperimmunglobulinemia; IgAN, IgA Nephropathy; Mo, Months; Yr, Years; NA, Data not available.

Accompanying disease was described in 9 patients (Table 2). An accompanying autoimmune disease (e. g. systemic lupus erythematosus, rheumatoid arthritis) is not uncommon in IPH patients [10,12]. Babbs et al. report a patient with IPH who developed IgA nephropathy and a nephrotic syndrome [17]. Splenectomy resulted in remission of the nephrotic syndrome. The cases presented by Noël et al. and Yoshimura et al. link IPH and renal transplantation; however, not on the basis of preexisting (autoimmune) renal disease [13,15]. The IPH case presented by Tsujimura et al. was associated with prominent polyclonal hyperimmunoglobulinemia and plasmacytosis in the bone marrow and the spleen, persisting even after splenectomy, and suggests an underlying chronic inflammatory state such as a connective tissue disease of the liver [16]. Association of various other diseases such as celiac disease [20], mixed connective tissue disease [21-25], and thyreoiditis [26-28] in IPH suggests that immunological disturbances are shared with autoimmune diseases [29], though exact mechanisms remain unclear.

### The Role of the Spleen in IPH Pathogenesis

In clearer cases of IPH, splenectomy turned out to be curative (median follow up 25 months, range 6-168 months, Table 2). The spleen seems to play a major role in the pathogenesis of IPH as disease remission can be observed after splenectomy in adults. It has been described that splenectomy or even splenic transposition may be an effective treatment of portal hypertension alone, and the reduction in the degree of flow thru the spleen into the portal circulation is expected to enhance liver function and reduce portal pressures post-splenectomy independent of any causality of the spleen and IPH. However, it is conceivable that the primary cause of IPH is not related to hepatic abnormalities but rather to abnormal splenic perfusion. Treska et al. described a young adult male with IPH and massive splenomegaly who was cured by splenectomy. The authors suggested, based upon immunohistochemical results, that the expression of VCAM-1 and ET-1 may be involved in splenic and portal hyperperfusion [7]. They found overexpression in IPH compared to healthy spleens. These molecules have been suggested to influence the development of IPH by causing hypercirculation and thereby hypertension in the portal venous system (ET-1) or by enhanced adhesion and migration of lymphocytes into the wall of the portal tracts followed by fibrosis (VCAM-1). Although the stimulus leading to augmented production of ET-1 and VCAM-1 in the enlarged spleen remains unknown, the circulating soluble isoforms of these factors may be elevated in the serum of IPH patients [7]. While VCAM-1 was also markedly elevated in our patient, we were unable to detect ET-1, either in serum or by immunohistochemistry. Sato et al. reported that sinus lining cells of IPH spleens showed diffuse and strong expression of inducible and endothelial nitric oxide synthetase (iNOS and eNOS). In contrast, ET-1 was detectable in only a few mononuclear leukocytes in the red pulp of IPH spleens [30]. These results suggest that NO liberated in spleen in the presence of low ET-1, is responsible for the dilatation of splenic sinuses, leading to splenomegaly and thereby contributing to portal hypertension in IPH [9]. This is intriguing, since our case represents the first case in which pathology underlying IPH was studied at such young age, making secondary phenomena less likely than in older patients with long-standing portal hypertension.

On this basis, VCAM-1 overexpression would not be a prime mechanism propagating portal hypertension but rather a secondary event that may be related to chronic vascular stress, inflammation, and phagocyte migration leading to nuclear factor *kappaB *(NF-kB) activation [31-33]. VCAM-1 expression may be induced in proliferating cells of myeloid origin in the spleen, which can either be a sign of extramedullary hematopoiesis or phagocytic activation [32]. The immunohistochemical staining in our case revealed that VCAM-1 was expressed not only in the endothelium but also in stromal cells. Instead, a marked expression of eNOS was found in splenic cells but not in the hepatic endothelium of our patient. However, the soluble form of VCAM-1 can be used for monitoring disease progression of IPH or the success of partial splenic embolization [34]. While an increase of adhesion molecules is a common phenomenon in cases of endothelial activation, the underlying pathophysiology of IPH seems to be different from arterial pulmonary hypertension of portopulmonary hypertension, in which and increased ET-1 expression and reduced NO lead to vasoconstriction [35,36].

## Conclusions

We report the first case of uncontrolled splenic hyperperfusion and enlargement with subsequent hypersplenism leading to life-threatening complications of IPH in infancy and show that disease remission after splenectomy in early childhood points to the spleen as the main organ underlying pathogenesis and progression of IPH. The success of surgical interventions targeting hypersplenism suggests that the primary defect in the regulation of splenic blood flow seems to be crucial for the development of IPH. This view of the pathophysiology is supported by the fact that i) liver function abnormalities do not occur in IPH, ii) abnormal eNOS and VCAM-1 expression was only seen in splenic but not hepatic tissue, iii) the signs of portal hypertension resolved in our patient after splenectomy, and iv) both the adult patients reported before and our young patient have not developed recurrent signs of portal hypertension, liver pathologies or complications after splenectomy. Thus, beside other treatment options (e. g. splenic embolization, percutaneous transhepatic obliteration or transjugular intraheptic portosystemic shunt procedure) total (or possible partial) splenectomy needs to be considered as a prime therapeutic option for IPH.

## Abbreviations

IPH: Idiopathic portal hypertension; eNOS: endothelial nitric oxide synthetase; VCAM-1: vascular endothelial adhesion molecule-1; ET-1: endothelin-1 (ET-1).

## Competing interests

The authors declare that they have no competing interests

## Authors' contributions

JD designed and performed the research, analyzed the data and wrote the paper; JW designed and performed the research and analyzed the data; UM performed magnetic resonance imaging of the abdomen, scintigraphic imaging with In-111-oxinate tagged, abdominal and Doppler ultrasound; BK performed splenectomy and liver biopsies; GK carried out histology and immunohistochemistry; CD cared for the patient and helped with analyzing date; MCF cared for the patient and helped with analyzing date; DF cared for the patient, designed and analyzed the research and helped with writing the paper. All authors read and approved the final manuscript.

## Pre-publication history

The pre-publication history for this paper can be accessed here:

http://www.biomedcentral.com/1471-230X/10/122/prepub

## Acknogledgements

We thank Kiran Nistala (University College London, Institute of Child Health, London, United Kingdom) and Trevelyan R. Menheniott (Murdoch Children’s Research Institute, Melbourne, Australia) for carefully reading the manuscript. Written informed consent was obtained from the patient’s parents for publication of this case report and any accompanying images. A copy of the written consent is available for review by the Editor-in-Chief of this journal.
